# LGBMDF: A cascade forest framework with LightGBM for predicting drug-target interactions

**DOI:** 10.3389/fmicb.2022.1092467

**Published:** 2023-01-05

**Authors:** Yu Peng, Shouwei Zhao, Zhiliang Zeng, Xiang Hu, Zhixiang Yin

**Affiliations:** School of Mathematics, Physics and Statistics, Shanghai University of Engineering Science, Shanghai, China

**Keywords:** drug-target interactions, machine learning, LightGBM, deep forest, prediction

## Abstract

Prediction of drug-target interactions (DTIs) plays an important role in drug development. However, traditional laboratory methods to determine DTIs require a lot of time and capital costs. In recent years, many studies have shown that using machine learning methods to predict DTIs can speed up the drug development process and reduce capital costs. An excellent DTI prediction method should have both high prediction accuracy and low computational cost. In this study, we noticed that the previous research based on deep forests used XGBoost as the estimator in the cascade, we applied LightGBM instead of XGBoost to the cascade forest as the estimator, then the estimator group was determined experimentally as three LightGBMs and three ExtraTrees, this new model is called LGBMDF. We conducted 5-fold cross-validation on LGBMDF and other state-of-the-art methods using the same dataset, and compared their Sn, Sp, MCC, AUC and AUPR. Finally, we found that our method has better performance and faster calculation speed.

## 1. Introduction

In recent years, with the rapid development of computer data processing capabilities, the continuous enrichment of data content, and the improvement of algorithm models, more and more researches on artificial intelligence in the fields of biology and medicine have been carried out (Guo et al., 2021; Chen and Yin, 2022; Zhou et al., 2022). Many computational methods based on machine learning have been proposed to solve biological problems (Lihong et al., 2021; Zhou et al., 2021; Peng et al., 2022; Shen et al., 2022). Especially in drug development, the prediction of drug-target interactions (DTIs) played an important role in drug development and drug repositioning, so using machine learning methods to predict DTIs became a research hotspot.

Over the past decade, a large number of machine learning-based methods were proposed for identifying DTI (Zhou et al., 2019). Among them, binary classification methods account for the majority. Some methods identify drug-target pairs based on drug and protein information, Li et al. (2020) used protein sequences and drug substructure fingerprint information to predict DTIs. In addition, there were many models (Mousavian et al., 2016; Li et al., 2020; Zhan et al., 2020; Tanoori et al., 2021) that predicted new DTIs based on information similarity.

In fact, there are more methods based on network inference, Yamanishi et al. (2010) integrated chemical, genomic and pharmacological information in bipartite graph to uncover potential DTIs. Mei J. P et al. (Mei et al., 2013) proposed Neighbor-based Interaction-profile Inferring (NII) based on bipartite local model (BLM). Chen et al. (2012) proposed the method of Network-based Random Walk with Restart on the Heterogeneous network (NRWRH) which integrates three different networks into a heterogeneous network through known DTIs, and achieves random wandering on this heterogeneous network. Cao et al. (2014) proposed a computational method for DTI prediction by combining the information from chemical, biological, and network properties. Ding et al. (2017) used molecular substructure fingerprints, multivariate mutual information (MMI) of proteins and network topology to represent drugs, targets and their relationships, and employ SVM and Feature Selection (FS) to build predictive models. Thereafter, scholars began to extract features from more complex networks. SNF-CVAE (Jarada et al., 2021) integrates similarity network fusion (SNF) and collective variational autoencoder (CVAE) to improve prediction accuracy. An and Yu (2021) proposed a Network Embedding framework in mulTiPlex networks (NEDTP) to predict DTIs. Jin et al. (2021) proposed a machine learning model called HeTDR, the method combines drug features in multiple networks and disease features in biomedical corpora to predict the degree of association between drugs and diseases. In addition, there are some computational methods based on matrix factorization (Gönen, 2012; Liu et al., 2016; Bagherian et al., 2021) and multi-label learning (Yuan et al., 2016; Pliakos et al., 2019; Chu et al., 2021b).

Moreover, with the rise of deep learning methods, people have made a lot of achievements in the field of DTI prediction based on deep learning methods. Many scholars consider graph analysis (Olayan et al., 2018; Peng et al., 2021; Yang et al., 2022) as an important means to predict DTIs. Many models apply deep neural networks (DNN) to DTI prediction, LASSO-DNN (You et al., 2019) combines LASSO with DNN, deepDTnet (Zeng et al., 2020b) applies DNN algorithm to network embedding, DeepFusionDTA (Pu et al., 2021) proposes a two-stage deep neural network ensemble model, based on DNN, DNN-DTIs (Chen et al., 2021) employs layer-by-layer learning method to predict DTIs. Besides, DeepACTION (Hasan Mahmud et al., 2020), AutoDTI++ (Sajadi et al., 2021), GCNMK (Wang et al., 2022) and DeepStack-DTIs (Zhang et al., 2022) also use deep learning methods.

Specially, inspired by DNN, Zhou and Feng (2017) proposed Deep Forest, and some DTI prediction methods based on Deep Forest showed good performance. Such as AOPEDF (Zeng et al., 2020a), DTI-CDF (Chu et al., 2021a) and EC-DFR (Lin et al., 2022).

In this study, we make some improvements based on the AOPEDF model, thus proposing a new method termed LGBMDF. We add LightGBM (Ke et al., 2017), which outperforms XGBoost and CatBoost in another work (Al Daoud, 2019), to Cascade Forest as a new estimator. For the convenience of comparison, we used the same feature extraction method as AOPEDF. For the obtained vector features, we input them into a modified Cascade Forest for predicting DTIs. Finally, we compared our model with other models in terms of performance and speed, our model is comparable to and in some way ahead of the state-of-the-art models. In conclusion, LGBMDF is a very practical method for DTI prediction, which can help new drug development and some other fields, such as identifying miRNA-disease associations or the associations between cancers and microbes.

## 2. Materials and methods

### 2.1. Data resource

DTI-related information was collected from DrugBank (v4.2) (Wishart et al., 2018), the Therapeutic Target Database (Yang et al., 2016), and the PharmGKB (Hernandez-Boussard et al., 2007) database. Bioactivity data for drug–target pairs are collected from ChEMBL (v20) (Gaulton et al., 2012), BindingDB (Liu et al., 2007), and IUPHAR/BPS Guide to PHARMACOLOGY (Pawson et al., 2014). The chemical structure of each drug with SMILES format is extracted from DrugBank (v4.0) (Law et al., 2014). Here, only DTIs meeting the following three criteria are used: (i) the human target is represented by a unique UniProt (Apweiler et al., 2004) accession number; (ii) the target is marked as ‘reviewed’ in the UniProt database; (iii) binding affinities, all the $\;K_{i},K_{d},IC50\;or\;EC50\leq 10\mu M$ . In short, we constructed a DTI network by using 732 FDA-approved drugs and 1915 targets. In addition, we used 9 drug-related networks and 6 protein-related networks (Cheng et al., 2019a,b; Zeng et al., 2020a). For the feature extraction approach, in order to facilitate comparison, we referred to the previous studies (Zhang et al., 2018; Zeng et al., 2020a).

### 2.2. Deep forest

The deep neural network has shown good performance in many works. Inspired by DNN, Zhou and Feng (2017) proposed an ensemble algorithm with deep structure based on decision tree. It has much fewer hyperparameters than DNNs, and the complexity of the model can be automatically determined based on the input variables.

**Figure 1:**
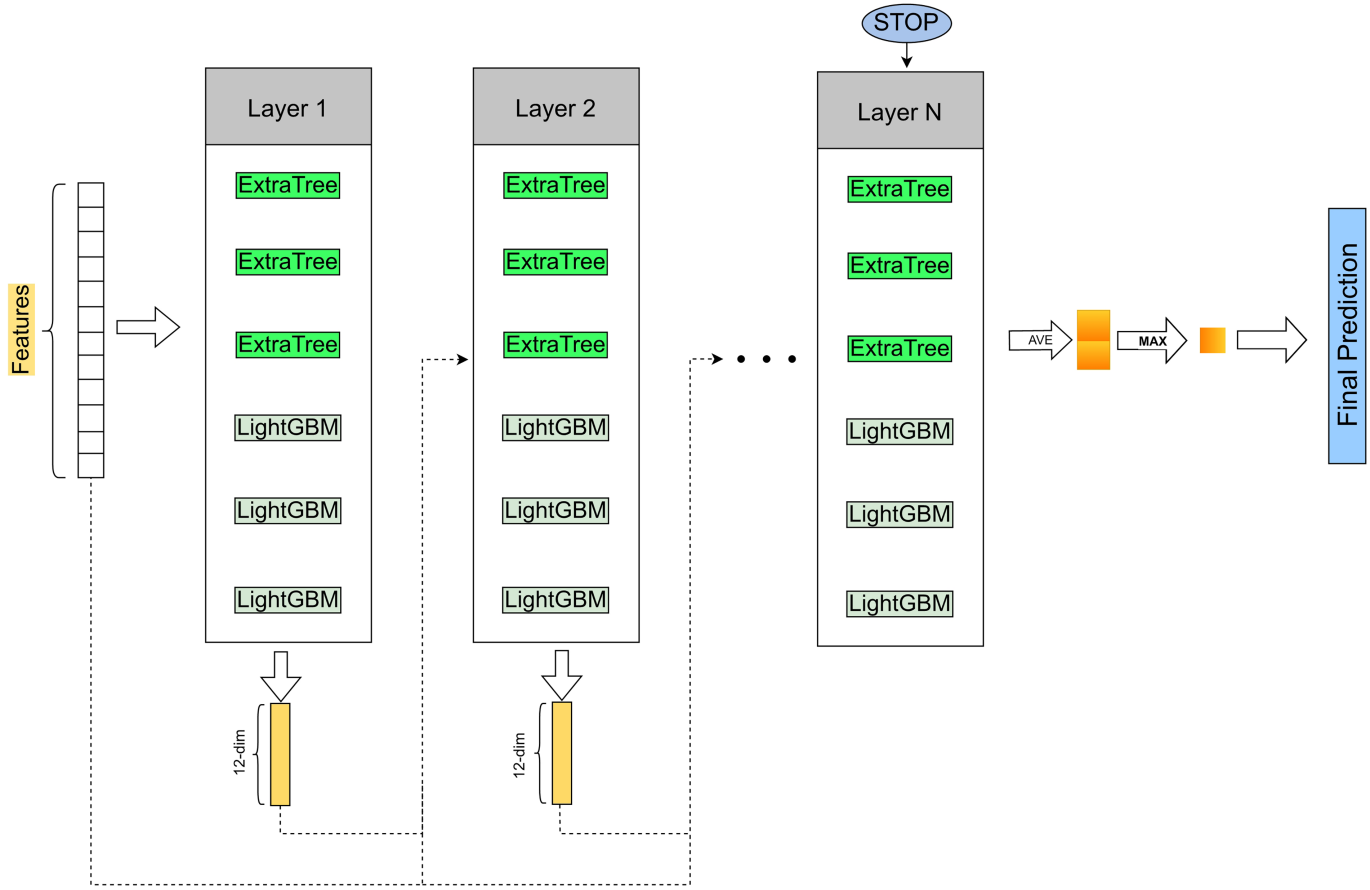
The pipeline of LGBMDF. After getting the features of drugs and targets, we process these features with cascade forest, and set 3 LightGBMs and 3 ExtraTrees for each level as estimators, each estimator outputs a 2-dimensional class vector, and then concatenate the output class vector and the original feature vector as the input vector for the next layer.

After obtaining low-dimensional vector representations of drugs and proteins (targets), we input them into Cascade Forest to predict DTIs. In the cascade structure, the output features vector of the previous layer and the original features vector is used as the input features vector of the next layer. Furthermore, when a new layer is generated, the performance of the entire cascade is estimated on the validation set, and the training process is terminated if there is no significant increase in performance. The estimators setting at each layer are also important, after experimental testing, we set up three ExtraTrees and three LightGBMs (Figure 1).

To prevent overfitting, class vectors for each estimator are generated by *k*-fold cross-validation. Specifically, the average of the generated *k*-1 class vectors is obtained to obtain the final class vector as the enhanced feature of the next layer.

### 2.3. LightGBM classifier

#### 2.3.1. Histogram algorithm

**Figure 2:**
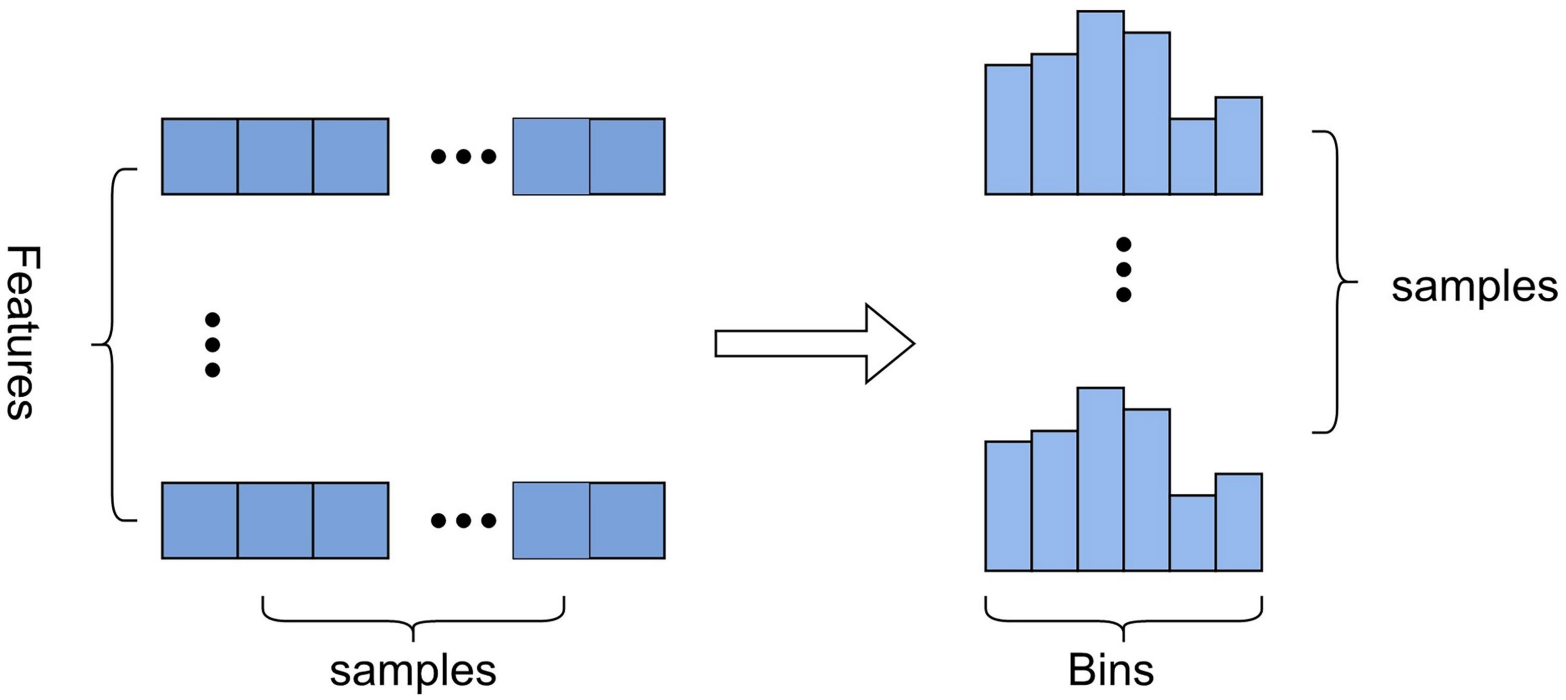
The construction of histogram.

The basic idea: First, the continuous floating-point feature values are discretized into k integers, and a histogram of width k is constructed (Figure 2). When the samples are traversed once, the histogram accumulates the required statistics and then traverses the histogram to find the optimal partition point based on the discrete values of the histogram.

**Figure 3:**
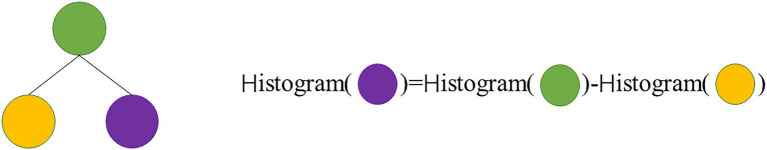
Subtract the histogram of sibling node from the histogram of the parent node so that the speed can be doubled.

Another improved speedup of LightGBM is to subtract the histogram of sibling nodes from the histogram of the parent node so that the speed can be doubled (Figure 3). Usually, when constructing a histogram, it is necessary to traverse all the data on that leaf, but histogram differencing only requires traversing k bins of the histogram. In the actual process of constructing the tree, LightGBM can also calculate the smaller leaf nodes of the histogram first, and then use histogram difference to obtain the larger leaf nodes of the histogram, so that we can get the histogram of its sibling leaf at a very small cost.

#### 2.3.2. Leaf-wise algorithm with depth restriction

**Figure 4:**
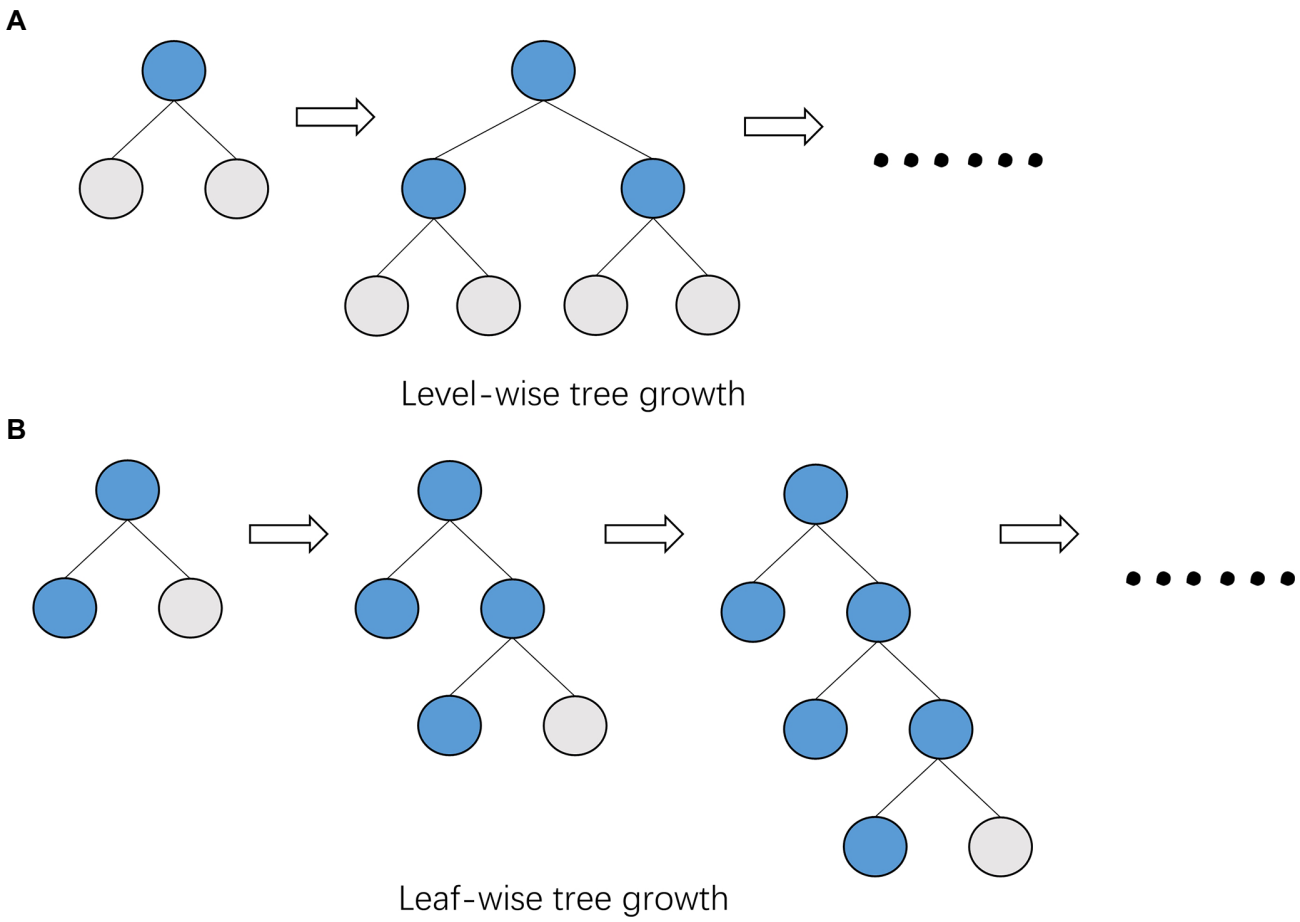
Comparison of tree growth patterns between XGBoost and LightGBM. **(A)** XGBoost uses the level-wise growth strategy, which can split the leaves of the same level at the same time by traversing the data once. **(B)** LightGBM uses the leaf-wise growth strategy, which finds the leaf with the largest splitting gain from all the current leaves, and then splits it.

Based on the histogram algorithm, LightGBM is further optimized. First, it abandons the level-wise (Figure 4A) tree growth strategy used by most GBDT algorithms and applies the leaf-wise tree growth (Figure 4B) with depth restriction. XGBoost uses level-wise growth strategy, which can split the leaves of the same level at the same time by traversing the data once, making it easy to perform multi-threaded optimization and control the model complexity without overfitting. However, level-wise is an inefficient algorithm because it treats the leaves of the same layer indiscriminately, and in fact, many leaves have low splitting gain, so there is no need to split, thus bringing a lot of unnecessary computational overhead. LightGBM uses leaf-wise tree growth strategy, which can locate the leaf with the largest splitting gain from all the current leaves, and then splits it, cycling as this way. Therefore, compared with level-wise, the advantage of leaf-wise is that it can reduce more errors and get better accuracy with the same number of splits; the disadvantage of leaf-wise is that it may grow a deeper decision tree and produce overfitting. For this reason, LightGBM adds a maximum depth limit to leaf-wise to ensure high efficiency and prevent overfitting at the same time.

#### 2.3.3. Gradient-based one-side sampling

The feature vector in Adaboost can represent the importance of a sample well, but there is not a weight vector like this one in GBDT. Fortunately, we found that the sample gradient of GBDT is a good indicator, and samples with small gradients will have small training errors and have been well-trained. Generally, the simpler idea is to discard samples with small gradients, but this will affect the model performance, thus we propose a new method named gradient-based one-side sampling (GOSS).

The basic idea of GOSS is to reduce the complexity of the model by reducing the sample size. GOSS first sorts the samples by the gradient from largest to smallest, uses the top-ranked a×100% , and then randomly samples the rest data with small gradients b×100% . Then GOSS amplifiers the data with a small gradient by a constant $\frac{1-a}{b}$ when calculating the information gain.

In GBDT, we assume the input space as $X^{s}$ , the gradient space as G . Suppose that there are n i.i.d instances $\left\{x_{1},x_{2},\cdots ,x_{n}\right\}$ , $x_{i}$ is a vector of dimension s in $X^{s}$ . The negative gradient of the loss function is represented as $\left\{g_{1},g_{2},\cdots ,g_{n}\right\}$ . The Decision tree model splits nodes where information gain is the largest, and the information gain is usually determined by the variance after the split.

Let O be the training set of a node d on the decision tree, and the variance of the split feature j at this point is defined as:


(1) $$V_{j\left|O\right.}\left(d\right)=\frac{1}{n_{O}}\left(\begin{array}{l} \frac{{\left(\sum_{\left\{x_{i}\in O:x_{ij}\leq d\right\}}g_{i}\right)}^{2}}{n_{l\left|O\right.}^{j}\left(d\right)}+\\ \frac{{\left(\sum_{\left\{x_{i}\in O:x_{ij}>d\right\}}g_{i}\right)}^{2}}{n_{r\left|O\right.}^{j}\left(d\right)} \end{array}\right)$$

Where $\begin{array}{l} n_{O}=\sum I\left[x_{i}\in O\right],n_{l|O}^{j}\left(d\right)=\sum I\left[x_{i}\in O:x_{ij}\leq d\right]\;\\ \;\;\;\;\;\;\;\;\;\;\;\;and\;n_{r|O}^{j}\left(d\right)=\sum I\left[x_{i}\in O:x_{ij}>d\right] \end{array}$ .

In GOSS, First, all instances absolute values of gradients are sorted in descending order. We select the first a×100% samples as set A , and then randomly sample B of size $b\times \#A^{c}|$ from the remaining instance set $A^{c}$ . Finally, we split the instance *via* estimated variance $\tilde{V_{j}}\left(d\right)$ on A∪B .


(2) $$\tilde{V_{j}}\left(d\right)=\frac{1}{n}\left(\begin{array}{l} \frac{{\left(\sum_{x_{i}\in A_{l}}g_{i}+\frac{1-a}{b}\sum_{x_{i}\in B_{l}}g_{i}\right)}^{2}}{n_{l}^{j}\left(d\right)}+\\ \frac{{\left(\sum_{x_{i}\in A_{r}}g_{i}+\frac{1-a}{b}\sum_{x_{i}\in B_{r}}g_{i}\right)}^{2}}{n_{r}^{j}\left(d\right)} \end{array}\right)$$

Where $\begin{array}{l} A_{l}=\left\{x_{i}\in A:x_{ij}\leq d\right\},A_{r}=\left\{x_{i}\in A:x_{ij}>d\right\},\\ B_{l}=\;\left\{x_{i}\in B:x_{ij}\leq d\right\},B_{r}=\left\{x_{i}\in B:x_{ij}>d\right\} \end{array}$ . $\frac{1-a}{b}$ is to normalize the size of B to the size of $A^{c}$ .

#### 2.3.4. Exclusive feature bundling

**Figure 5:**
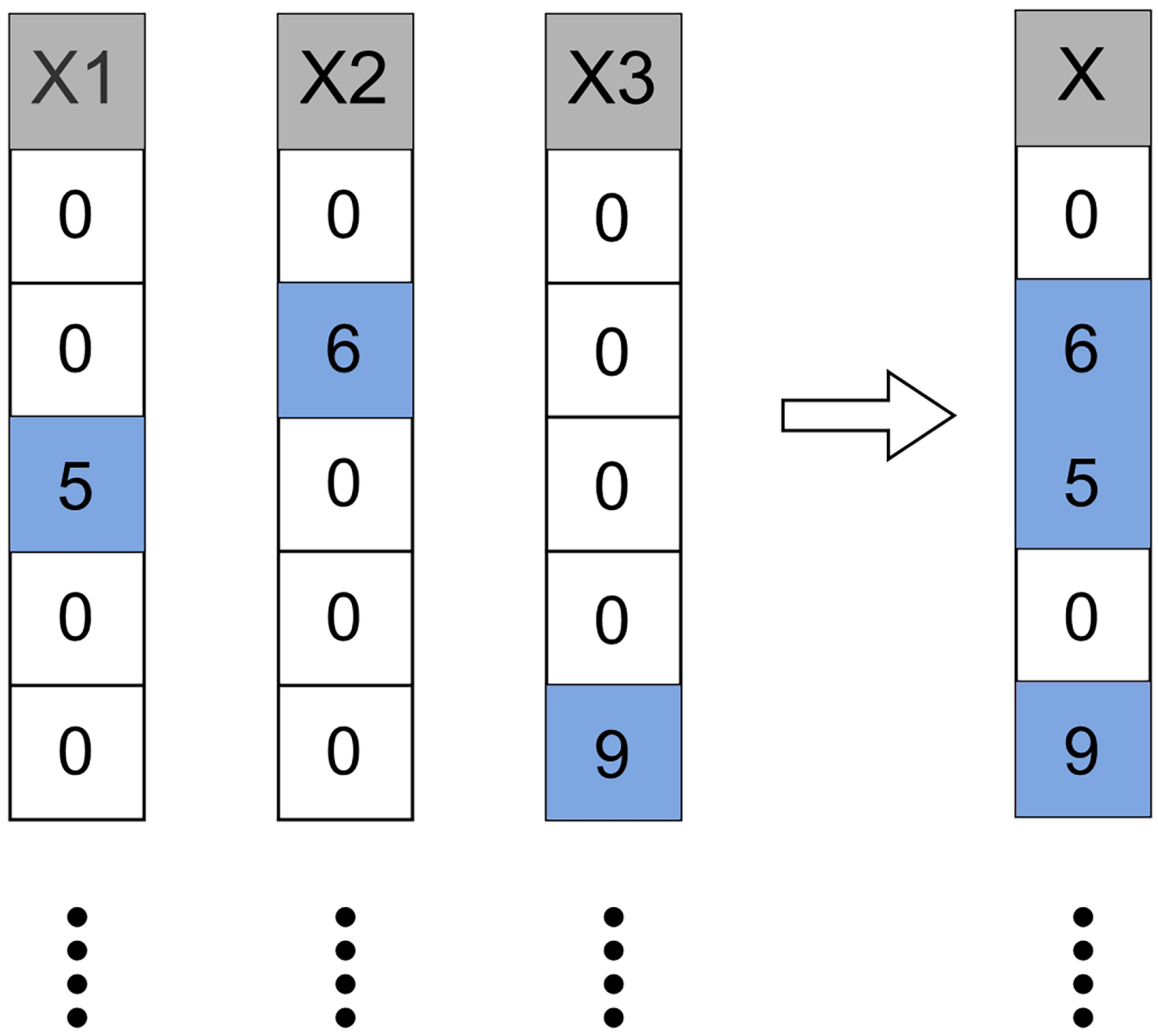
Bind mutually exclusive features into a single feature.

High-dimensional space is always sparse, and in a sparse feature space, many features are mutually exclusive, so we can bind mutually exclusive features into a single feature (Figure 5). Through the feature scanning algorithm, we can use the designed feature scanning algorithm to construct the same histogram from the feature bundles as the original single feature. In this way, we can decrease the complexity of histogram building from O(#sample×#feature) to O(#sample×#bundle) , while #bundle≪#feature , thus we can greatly improve the training speed of GBDT.

In general, compare to XGBoost, LightGBM has the advantages of faster speed and smaller memory usage. LightGBM uses the histogram algorithm to transform the traversal samples into traversal histograms, which greatly reduces the time complexity; applies the GOSS algorithm to filter out many samples with small gradients and adopts leaf-wise growth strategy to build the trees, which reduces a lot of unnecessary calculations. In addition, LightGBM utilizes EFB algorithm to decrease the number of features.

### 2.4. Evaluation metric

To compare with other methods, we perform a 5-fold cross-validation and adopt Sn, Sp, MCC, AUC and AUPR as evaluation metrics.

Sn, Sp and MCC are commonly used evaluation indicators for binary classification problems, and their calculations are based on the confusion matrix.


(3) $$S_{n}=\frac{TP}{TP+FN}$$

(4) $$S_{p}=\frac{TN}{TN+FP}$$

(5) $$MCC=\frac{TP\times TN-FP\times FN}{\sqrt{\left(TP+FP\right)\left(TP+FN\right)\left(TN+FP\right)\left(TN+FN\right)}}$$

Receiver operating characteristic (ROC) curve is often used to evaluate the model’s prediction performance. It is calculated based on the confusion matrix. The higher the curve on the upper left, the better the performance of the model. The vertical axis of the ROC curve is the “True Positive Rate,” and the horizontal axis is the “False Positive Rate,” which are, respectively, defined as:


(6) $$TPR=\frac{TP}{TP+FN}$$

(7) $$FPR=\frac{FP}{TN+FP}$$

However, the ROC curves of some models will cross, so we generally choose the AUC (Area Under ROC Curve) for comparison. We assume that the points of the ROC curve are connected in order by the points of $\left\{\left(x_{1},y_{1}\right),\left(x_{2},y_{2}\right),\cdots ,\left(x_{m},y_{m}\right)\right\}$ , then the AUC can be estimated as:


(8) $$AUC=\frac{1}{2}\overset{m-1}{\underset{i=0}{\sum }}\left(x_{i+1}-x_{i}\right)•\left(y_{i+1}-y_{i}\right)$$

The PR curve represents the relationship between Precision and Recall. In general, Recall is set to the abscissa and Precision is set to the ordinate. Precision and Recall can be calculated according to the confusion matrix.


(9) $$Precision=\frac{TP}{TP+FP}$$

(10) $$Recall=\frac{TP}{TP+FN}$$

AUPR is the Area Under PR curve. In such a highly imbalanced dataset, AUPR can provide better performance evaluation because it penalizes false positives more severely.

## 3. Results

### 3.1. Parameter optimization

We optimized the parameters of the estimators, considering the impact of parameters on model performance. By the means of employing GridSearchCV function, we set the interval of the parameter, the “scoring” is set as “accuracy.” The parameter optimization results are shown in Table 1.

**Table 1:** The result of parameter optimization.

Model	Parameter	Range	Used
RandomForest	n_estimators	[100, 200, 400, 500, 600]	400
LightGBM	n_estimators	[100, 200, 400, 500]	400
	max_depth	[7, 8, 9, 10, 11]	11
	num_leaves	[100, 200, 300, 400, 500]	200
ExtraTree	n_estimators	[100, 200, 400, 500, 600]	500

### 3.2. Estimators setting for each layer

When reproducing the AOPEDF model, we noticed that the XGBoost in cascade is time-consuming, so we chose LightGBM, a classifier that performs better than XGBoost in another work (Al Daoud, 2019), as estimator to accelerate the calculation speed of the model and reduce the computing cost and time cost. We tested five combinations and compared their Sn, Sp, MCC, AUC, AUPR (Table 2) and running time. The experiments are run in the environment of Python3.9, CPU: 2* Intel (R) Xeon (R) Gold 6320R, RAM: 128G.

**Table 2:** Performance comparison under each estimator setting.

Estimators	Sn	Sp	MCC	AUC	AUPR
AOPEDF	**0.9463**	0.9447	0.8911	0.9842	0.9855
2LGB-2RF-2ET	0.9439	**0.9477**	0.8918	0.9841	0.9854
3LGB-3RF	0.9443	0.9453	0.8898	0.9839	0.9849
3LGB-3ET	0.9451	0.9471	**0.8924**	**0.9844**	**0.9857**

The bold values represent the maximum value of each estimator setting under each evaluation metric.

**Figure 6:**
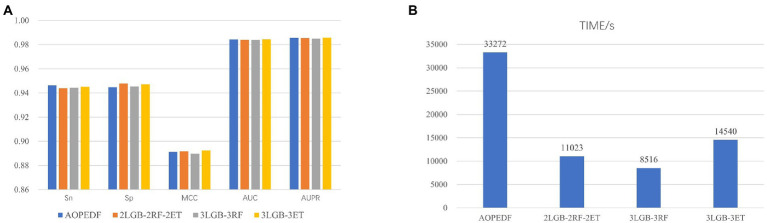
Model performance comparison under each estimator setting. **(A)** AUC and AUPR for 4 estimator combinations. **(B)** Computational time for 4 estimator combinations.

The names of each combination in the Figure 6 are explained as follows:

- *AOPEDF*: 2 ExtraTrees, 2 RFs and 2 XGBoosts
- *2LGB-2RF-2ET*: 2 LightGBMs, 2 RFs and 2 ExtraTrees
- *3LGB-3RF*: 3 LightGBMs and 3 RFs
- *3LGB-3ET*: 3 LightGBMs and 3 ExtraTrees.

After experiments, we found that the MCC, AUC and AUPR values of 3LGB-3ET are higher than that of the others. Moreover, the calculation speed of 3LGB-3ET is more than twice as fast as AOPEDF. Therefore, we choose the combination of 3LGB-3ET to set the estimators for each layer finally.

### 3.3. Model comparison

The following 4 models were adopted as baseline methods.

NEDTP (An and Yu, 2021): A node similarity network is constructed based on 15 heterogeneous information networks, and then random walks are applied to extract the topology information of each node in the network and learn it as a low-dimensional vector. Finally, employ LightGBM algorithm to complete the classification task.

AOPEDF (Zeng et al., 2020a): It integrates 15 biological networks to construct a heterogeneous network, and then learns low-dimensional vector representations of features from this heterogeneous network that keep arbitrary-order proximity. Then use the deep forest to predict new DTIs.

Random Forest (Breiman, 2001): It is a combination of tree predictors such that each tree depends on the value of an independently sampled random vector and all trees in the forest have the same distribution.

Support Vector Machine, SVM (Vapnik and Chervoneva, 1964): It is a class of generalized linear classifiers for binary classification of data in a supervised learning manner.

**Figure 7:**
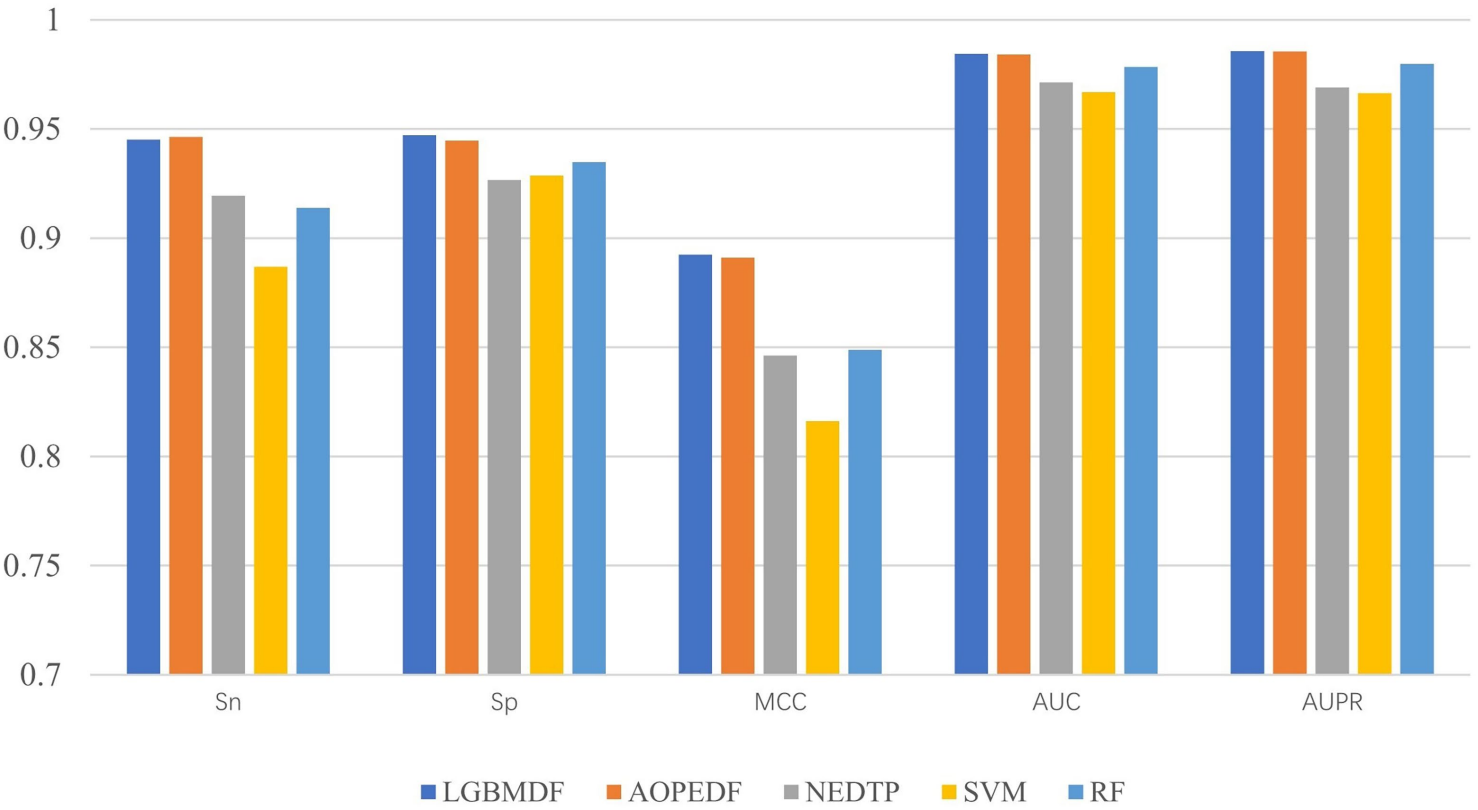
Sn, Sp, MCC, AUC and AUPR of LGBMDF, AOPEDF, NEDTP, RF, SVM.

We took drug-protein pairs with known interactions as positive samples, and pairs with unknown interactions as negative samples, and then selected all positive samples and randomly sampled negative samples with the same number of positive samples for 5-fold cross-validation to evaluate model performance (Figure 7, Table 3). For each 5-fold cross-validation, we select 80% positive pairs and the corresponding number of randomly sampled negative pairs as the training set, and the remaining 20% positive pairs and the corresponding number of randomly sampled negative pairs as the test set. We found that the Sp, MCC, AUC, and AUPR of LGBMDF are all higher than those of other methods. In addition, in previous experiments, we have found that LGBMDF is faster than AOPEDF. An excellent model needs to consider both the accuracy and the computing power cost of the model. Therefore, our model is better than the current advanced model in general.

**Table 3:** Performance of LGBMDF and baseline methods.

Model	Sn	Sp	MCC	AUC	AUPR
LGBMDF	0.9451	**0.9471**	**0.8924**	**0.9844**	**0.9857**
AOPEDF	**0.9463**	0.9447	0.8911	0.9842	0.9855
NEDTP	0.9194	0.9267	0.8462	0.9714	0.9690
SVM	0.8869	0.9286	0.8162	0.9668	0.9664
RF	0.9138	0.9348	0.8488	0.9784	0.9798

The bold values represent the maximum value of each estimator setting under each evaluation metric.

## 4. Discussion

This paper investigated the application of machine learning methods for DTI prediction. Traditional drug-target effect testing methods are time-consuming and labor-intensive. And Machine learning methods have attracted the attention of many researchers due to these methods can greatly reduce the related costs. We chose the same feature extraction method as AOPEDF, and used this method to extract low-dimensional representations of drug and protein features from 15 biological networks, and these features maintain arbitrary order proximity.

After obtaining low-dimensional feature representations of drugs and targets, we used cascaded deep forests for DTI prediction. Specifically, we used LightGBM as the estimator in the cascade to reduce the computational cost. And the LightGBM has shown better performance and computational speed than XGBoost in other experiments. Considering the effect of estimator diversity in the cascade, we also chose ExtraTree as the estimator. By comparing the Sn, Sp, MCC, AUC, AUPR and computation time of the 4 estimator combinations, we chose three ExtraTrees and three LightGBMs as estimators at each layer, and then utilized this cascade forest for DTI prediction. To demonstrate the merits of our model, we compared it with other four baseline models on the same dataset. After 5-fold cross-validation, we obtained the Sn, Sp, MCC, AUC and AUPR of the five models, the Sp (0.9471), MCC (0.8924), AUC (0.9844) and AUPR (0.9857) of LGBMDF were higher than AOPEDF, NEDTP, RF and SVM. The Sn (0.9451) was slightly inferior to AOPEDF, but higher than other three methods. Furthermore, the calculation time of LGBMDF was less than half of that of AOPEDF.

In summary, the method proposed in this paper shows higher prediction accuracy with the current state-of-the-art methods, and greatly improves the computational speed. We believe this will accelerate the drug development process to a certain extent. Certainly, there are still some shortcomings in this paper, such as feature extraction method. We believe that if there is a better way to extract features, the prediction accuracy will also be improved. Moreover, our method could also be applied in other studies, such as in exploring the link between microbes and cancer.

## Data availability statement

The data and code for LGBMDF is available at https://github.com/TLanCZ/LGBMDF.

## Author contributions

YP proposed the model and completed the manuscript writing. ZZ and XH assisted in completing the model construction. SZ and ZY reviewed and revised the manuscript. ZY provided financial support. All authors contributed to the article and approved the submitted version.

## Funding

This research was supported by National Natural Science Foundation of China (no: 62072296).

## Conflict of interest

The authors declare that the research was conducted in the absence of any commercial or financial relationships that could be construed as a potential conflict of interest.

## Publisher’s note

All claims expressed in this article are solely those of the authors and do not necessarily represent those of their affiliated organizations, or those of the publisher, the editors and the reviewers. Any product that may be evaluated in this article, or claim that may be made by its manufacturer, is not guaranteed or endorsed by the publisher.
